# Constructing a screening model to obtain the functional herbs for the treatment of active ulcerative colitis based on herb-compound-target network and immuno-infiltration analysis

**DOI:** 10.1007/s00210-023-02900-z

**Published:** 2023-12-20

**Authors:** Haiya Ou, Xiaopeng Ye, Hongshu Huang, Honghui Cheng

**Affiliations:** Shenzhen Bao’an Traditional Chinese Medicine Hospital, Guangzhou University of Chinese Medicine, Shenzhen, China

**Keywords:** Traditional Chinese Medicine (TCM) prediction, Ulcerative colitis (UC), Machine learning, Immuno-infiltration analysis, Weighted gene co-expression network analysis (WGCNA)

## Abstract

The therapeutic effect of most traditional Chinese medicines (TCM) on ulcerative colitis is unclear, The objective of this study was to develop a core herbal screening model aimed at facilitating the transition from active ulcerative colitis (UC) to inactive. We obtained the gene expression dataset GSE75214 for UC from the GEO database and analysed the differentially expressed genes (DEGs) between active and inactive groups. Gene modules associated with the active group were screened using WGCNA, and immune-related genes (IRGs) were obtained from the ImmPort database. The TCMSP database was utilized to acquire the herb-molecule-target network and identify the herb-related targets (HRT). We performed intersection operations on HRTs, DEGs, IRGs, and module genes to identify candidate genes and conducted enrichment analyses. Subsequently, three machine learning algorithms (SVM-REF analysis, Random Forest analysis, and LASSO regression analysis) were employed to refine the hubgene from the candidate genes. Based on the hub genes identified in this study, we conducted compound and herb matching and further screened herbs related to abdominal pain and blood in stool using the Symmap database.Besides, the stability between molecules and targets were assessed using molecular docking and molecular dynamic simulation methods. An intersection operation was performed on HRT, DEGs, IRGs, and module genes, leading to the identification of 23 candidate genes. Utilizing three algorithms (RandomForest, SVM-REF, and LASSO) for analyzing the candidate genes and identifying the intersection, we identified five core targets (CXCL2, DUOX2, LYZ, MMP9, and AGT) and 243 associated herbs. Hedysarum Multijugum Maxim. (Huangqi), Sophorae Flavescentis Radix (Kushen), Cotyledon Fimbriata Turcz. (Wasong), and Granati Pericarpium (Shiliupi) were found to be capable of relieving abdominal pain and hematochezia during active UC. Molecular docking demonstrated that the compounds of the four aforementioned herbs showed positive docking activity with their core targets. The results of molecular dynamic simulations indicated that well-docked active molecules had a more stable structure when bound to their target complexes. The study has shed light on the potential of TCMs in treating active UC from an immunomodulatory perspective, consequently, 5 core targets and 4 key herbs has been identified. These findings can provide a theoretical basis for subsequent management and treatment of active UC with TCM, as well as offer original ideas for further research and development of innovative drugs for alleviating UC.

**Supplementary Information:**

The online version contains supplementary material available at 10.1007/s00210-023-02900-z 10.1007/s00210-023-02900-z.

## Introduction

Ulcerative colitis (UC) is a prevalent form of inflammatory bowel disease (IBD) that is characterized by recurrent and diffuse inflammation in the rectal and colonic mucosa, with abdominal pain and hematochezia as principal clinical symptoms. In recent decades, the incidence of UC has rapidly increased (Du and Ha 2020).. Despite the high prevalence, the pathogenesis of UC remains uncertain, it is believed that genetic and environmental factors as well as immune system dysfunction can disrupt the progressive repair-damage equilibrium of the intestinal mucosa and thus contribute to the pathogenesis (Piovani et al. 2019; Ray and Longworth 2019). The primary treatment objective for UC is to maintain steroid-free remission (Rubin et al. 2019), Treatment with 5-aminosalicylic acid (5-ASA), salazosulfapyridine (SASP), corticosteroids, thiopurines and molecularly targeted drugs is currently used depending on the severity. Among those, 5-ASA is the preferred choice for mild to moderate UC (Le Berre et al. 2020). Nevertheless, about 11% of the patients are intolerant to mesalazine and may experience discomfort such as increased diarrhea, fever, and abdominal pain (Hiraoka et al. 2021). Besides, the interruption of 5-ASA therapy due to intolerance may lead to adverse clinical outcomes such as enterectomy and bowel cancer (Hiraoka et al. 2021). However, long-term administration of SASP is believed to result in infertility, as well as damage on kidney and liver (Linares et al. 2011). Therefore, it is crucial to explore a safer and more effective therapeutical methods to achieve sustained remission for UC.

Immune dysregulation is the primary factor in the pathogenesis of UC. The immune response and inflammatory pathways of UC are driven by dynamic and complex interactions of immune cells and cytokines. Studies indicate that during active periods of UC, Treg cells significantly proliferate, and the balance between Th17 and Treg cells is critical in the T cell-mediated immune response in the gut. Restoring the balance of Treg/Th17 cells and reconstructing the gut microbiota is a promising and novel therapeutic target for UC treatment (Himmel et al. 2012; Zhu et al. 2020). Furthermore, administering the polarization of macrophage M1/M2 to retain immune homeostasis is also a valuable therapeutic strategy to induce UC remission (Wu et al. 2021). In addition, antigen-presenting cells (dendritic cells and macrophages), helper T cells, regulatory T cells, and natural killer T cells have been verified to play a crucial role in the pathogenesis of UC. Meanwhile, neutrophils, natural killer (NK) cells, and several cytokines including tumor necrosis factor A (TNF-A), interferon G (IFN -G), IL17A, CD161, CD56, and IL-22 are found infiltrating into the intestinal mucosa of UC patients in large numbers (Lim et al. 2016; Mitsialis et al. 2020). In conclusion, these findings suggest that immunomodulation is a crucial part of the pathogenesis of UC, as well as a vital therapeutical target.

**Fig. 1:**
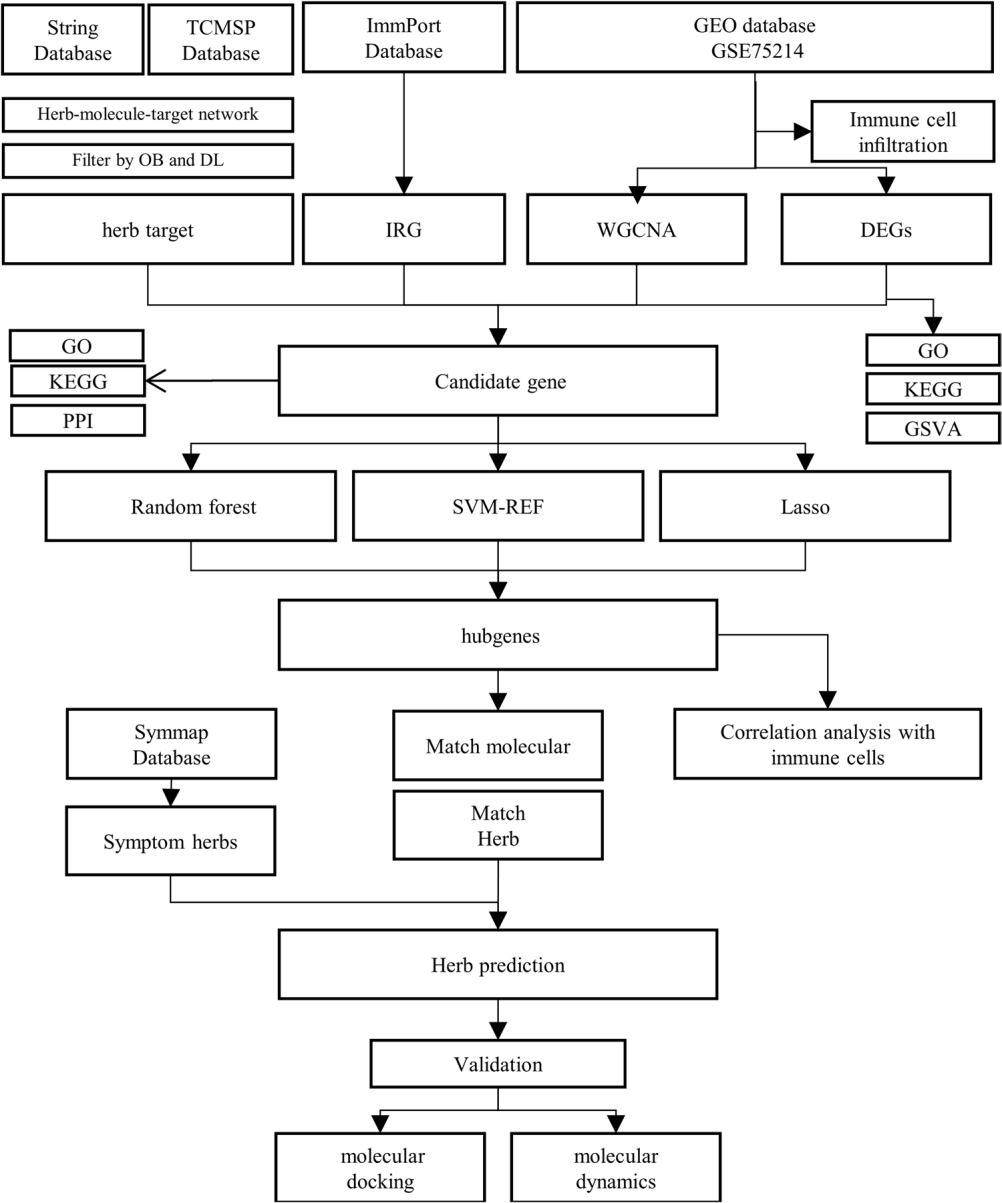
The flow chart of the present study

It is evidenced that certain kinds of Traditional Chinese Medicines (TCMs) are effective and safe for the therapy of UC (Chen et al. 2020). Researches has shown that TCM monomers such as Juglone (Hua et al. 2021) and Baicalin, as well as herbal formula like Pulsatilla Decoction and Black Plum Pills (Miao et al. 2020; Yan et al. 2022), have the capability to regulate immune balance of Treg/Th17 cells and polarization of macrophages M1/M2, and to reduce mucosal injury of the colon. These findings suggest the potential of TCM for preventing and alleviating UC in clinical settings. In addition, TCM is composed of multiple components, with multiple targets and pathways of working, making it a predominant solution for diseases with complex pathogenesis. However, it is still unclear whether most TCMs can modulate UC immune function, and further exploration is necessary. Based on the analysis above, we investigated the differences in immune cell infiltration and immune-related gene expression values between active and inactive UC. Then we screened out the core targets of regulating the UC immune mechanism by TCMs based on machine learning method. The molecules or TCMs which is capable of treating UC was screened out via constructing a target-compound-herbs network. Finally, we validated the activity of these molecules with the help of computational pharmacology such as molecular docking and molecular dynamic simulation. The study aims to provide a theoretical basis for proposing an original TCM solution for UC in clinical settings, as well as provide innovative ideas for the research and development of novel drug for the therapy of UC. The research flow chart of this study is shown in Fig. 1.

## Methods

### Identification of differentially expressed genes of UC via gene expression profiles

The dataset GSE75214 from the Gene Expression Omnibus (GEO) database was chosen for analysis (Vancamelbeke et al. 2017), which contains 23 samples of inactive UC colonic mucosal tissue and 74 samples of active UC colonic mucosal tissue. Differential expression analysis of genes between the active and inactive groups was performed by the help of the Limma package (Ritchie et al. 2015). All probe IDs were converted to gene symbols, and differentially expressed genes (DEGs) identified with the cut-off of p < 0.05 and |log Fc|≥ 1.

### GO, KEGG pathway and GSEA enrichment analysis

Using the "ClusterProfiler" and "Enrichplot" package, Gene ontology (GO) and Kyoto Encyclopedia of Genomes (KEGG) pathway enrichment analysis of DEGs was performed with a significance level set at p ≤ 0.05 and q ≤ 0.05 (Yu et al. 2012). To explore the underlying immune response mechanisms in transition from an inactive to an active state, immunological signature gene sets from the Molecular Signature Database (MsigDB) (Liberzon et al. 2015) were utilized for Gene Set Enrichment Analysis (GSEA). Significantly enriched gene sets were identified with p < 0.05 and false discovery rate (FDR) < 0.05.

### Acquisition of immune-related genes and immune infiltration analysis

One thousand eight hundred and eleven immune-related genes (IRGs) were obtained from the immune-related datasets which were acquired from the IMMPORT database (Bhattacharya et al. 2014). The relative levels of immune cell infiltration in 97 samples were quantified by the Cibersort inverse fold method with the help of the "IOBR" package (Zeng et al. 2021).

### Construction of gene co-expression network and access to key module genes

The gene expression matrix used in this analysis consisted of the top 3000 genes with the largest standard deviations from dataset GSE75214. To generate a co-expression network, the Weighted Gene Co-expression Network Analysis (WGCNA) package in R software was employed (Langfelder and Horvath 2008). The soft threshold was determined by an R^2 value close to 0.85, allowing for the construction of a scale free network. Additionally, a topological overlap matrix (TOM) was created from the adjacency matrix. Using a dynamic tree cutting algorithm, gene modules were identified to pinpoint the genes involved in active and inactive phases.

### Construction of herb-molecule-target network and acquisition of therapeutic targets of TCM

The correspondence among the herb-molecule-target was collected from the Traditional Chinese Medicine Systems Pharmacology (TCMSP) database (http://tcmspw.com/) (Ru et al. 2014). To create the herb-molecule-target network, only those with an oral bioavailability (OB) of ≥ 30% and drug likeness (DL) of ≥ 0.18 were determined to be herb-molecule-targets. Additionally, the targets within the network are known as herb-related targets (HRTs). Subsequently, the network was disassembled into three sub-networks including herb-target network, molecule-target network and herb-molecule network for further analysis. Finally, with the assistance of the String database (https://cn.string-db.org/), all protein names of HRTs from the network were converted into gene symbols.

### Acquisition and functional enrichment analysis of candidate genes related to immune regulation by TCM

Candidate genes regarding treating active ulcers by TCM through immune regulation are those included in all DEGs, HRT, crucial modular genes in co-expression network, and IRG. The potential functional mechanism of candidate genes was elaborated by analyzing protein–protein interaction networks (PPI) using the String database and performing enrichment analysis of candidate genes through GO and KEGG (threshold: p ≤ 0.05 and q ≤ 0.05).

### Screening hub genes from candidate genes via machine learning algorithms

To identify core targets, we selected the expression matrix containing the **candidate genes** from the GSE75214 dataset. Subsequently,"e1071" (Meyer et al. 2015), "randomFores" package (Liaw and Wiener 2002) and "glmnet" package (Friedman et al. 2010) are used respectively to perform support vector machine-recursive feature elimination (SVM-RFE), Random Forest and LASSO regression analysis to further screening of hub targets from the candidate genes.

SVM-RFE is a gene selection method that employs the Support Vector Machine technique based on Recursive Feature Elimination (RFE). It iteratively eliminates the least effective features from the model, and then retrains the model using the remaining features to generate an improved and more concise subset, thereby achieving optimal model performance (Guyon et al. 2002).Consequently, the SVM-RFE algorithm is utilized for identifying genes with heightened discriminative power (Lin et al. 2017; Huang et al. 2018). In this study, SVM-RFE was employed to ascertain the optimal subset of feature genes utilizing tenfold cross-validation.

The gene expression matrix of candidate genes is utilized as an input data for analysis in a random forest model.The model utilizes 500 decision trees. The analysis aims to determine if the misclassification rate stabilizes as the number of decision trees in the forest increases.After the rate stabilizes, the impact of each gene on the results will be analyzed, and the top 15 most significant genes will be selected.

The Lasso regression achieves sparsity by incorporating a penalty term (L1 regularization) into the optimization objective function, resulting in many feature weights in the coefficient vectors becoming zero. We further conducted LASSO regression analysis and applied tenfold cross-validation to the candidate gene matrix to identify core targets by selecting the features corresponding to non-zero coefficients.

These intersection genes, identified by the three machine learning algorithms, are considered core targets for promoting remission of active UC through regulation of immune reactions by TCM.

### Screening functional herbs based on hub genes

Hub genes is used to screen sub-networks from the herb-target and compound-target networks, so as to identify herbs and molecules related to those hub genes. To identify herbs with the potential to treat active UC by regulating immune reactions and relieving clinic symptoms, the "abdominal pain"-related and "hematochezia"-related herbs were obtained from the Symmap database (https://www.symmap.org/) (Wu et al. 2019), which were intersected with the hub target-related herbs to identify the relevant candidates.

### Molecular docking validation

Molecules related to the herbs were extracted from the compound-target network. The structure files of the molecules were acquired from the TCMSP database and the crystal structures of the core protein targets were obtained from the PDB database (https://www.rcsb.org/pdb). Subsequently, molecular docking was performed using Autodock Vina (Trott and Olson 2010). Besides, the binding energy of the complexes were obtained to assess the binding mode and the structural stability among the complexes, binding activity is considered good if Affinity < -5.0 kcal/mol, and strong if Affinity ≤ -7.0 kcal/mol (Trott and Olson 2010). The visualization of receptor-ligand binding conformation and acting force was achieved via the Protein–Ligand Interaction Profiler(HIPL) website (https://plip-tool.biotec.tu-dresden.de) (Salentin et al. 2015) and Pymol 1.7 software. Active components and targets with good binding activity were identified with Affinity.

### Molecular dynamic simulation

Molecular dynamic simulation of the molecular-protein complex with the lowest Affinity is performed using Gromacs 5.1.4 to analyze the interaction between the target proteins and the molecules (Van Der Spoel et al. 2005). The protein topological parameters were derived from the Gromos96 54a7 force field. For preparation of the simulation, the complex was placed in a unit box (parameters: -bt dodecahedron;—d 1.0); subsequently, procedures including ion addition, energy minimization and pre-equilibration were performed; finally, molecular dynamic simulation was performed at 26.85 °C (300 K) for 50 ns. The root mean square deviation (RMSD) of the complexes, and root mean square fluctuations (RMSF) of the residues was calculated to assess the stability of the complexes.

## Result

### Identification of DEGs between active and inactive UC group

**Fig. 2:**
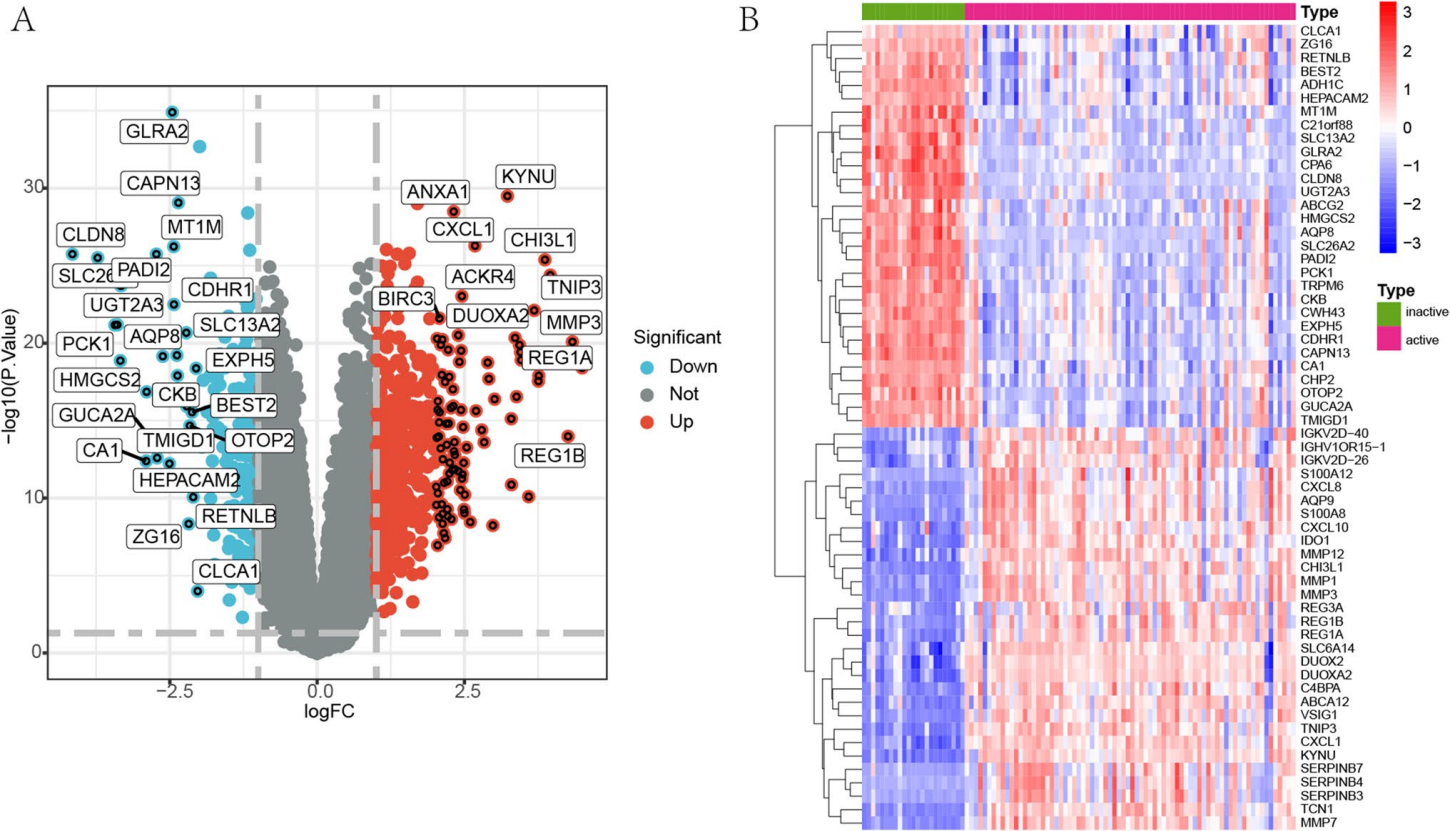
Differentially expressed genes between active and inactive UC. **A** Volcano plot: red represents up-regulated genes; blue represents down-regulated genes. **B** Top 30 up-regulated and down-regulated genes. Genes in red represent up-regulation and genes in blue represent down-regulation

To gain a comprehensive understanding of the gene expression profiles in active and inactive UC group, the GSE75214 dataset was normalized and then differential analysis of the genes was performed between the two groups. A total of 729 differentially expressed genes were identified, including 527 up-regulated genes (e.g. VSIG1, SERPINB3, DUOXA2, MMP1, S100A8, CHI3L1, TNIP3, REG1B, MMP3, REG1A) and 202 down-regulated genes (e.g. CLDN8, SLC26A2, PCK1, AQP8, HMGCS2, UGT2A3, CA1, ADH1C, GUCA2A, PADI2). Figure 2-A shows the volcano plot of DEGs for the dataset, and Fig. 2-B displays the top 30 up-regulated and down-regulated genes.

### GO and KEGG pathway enrichment analysis

**Fig. 3:**
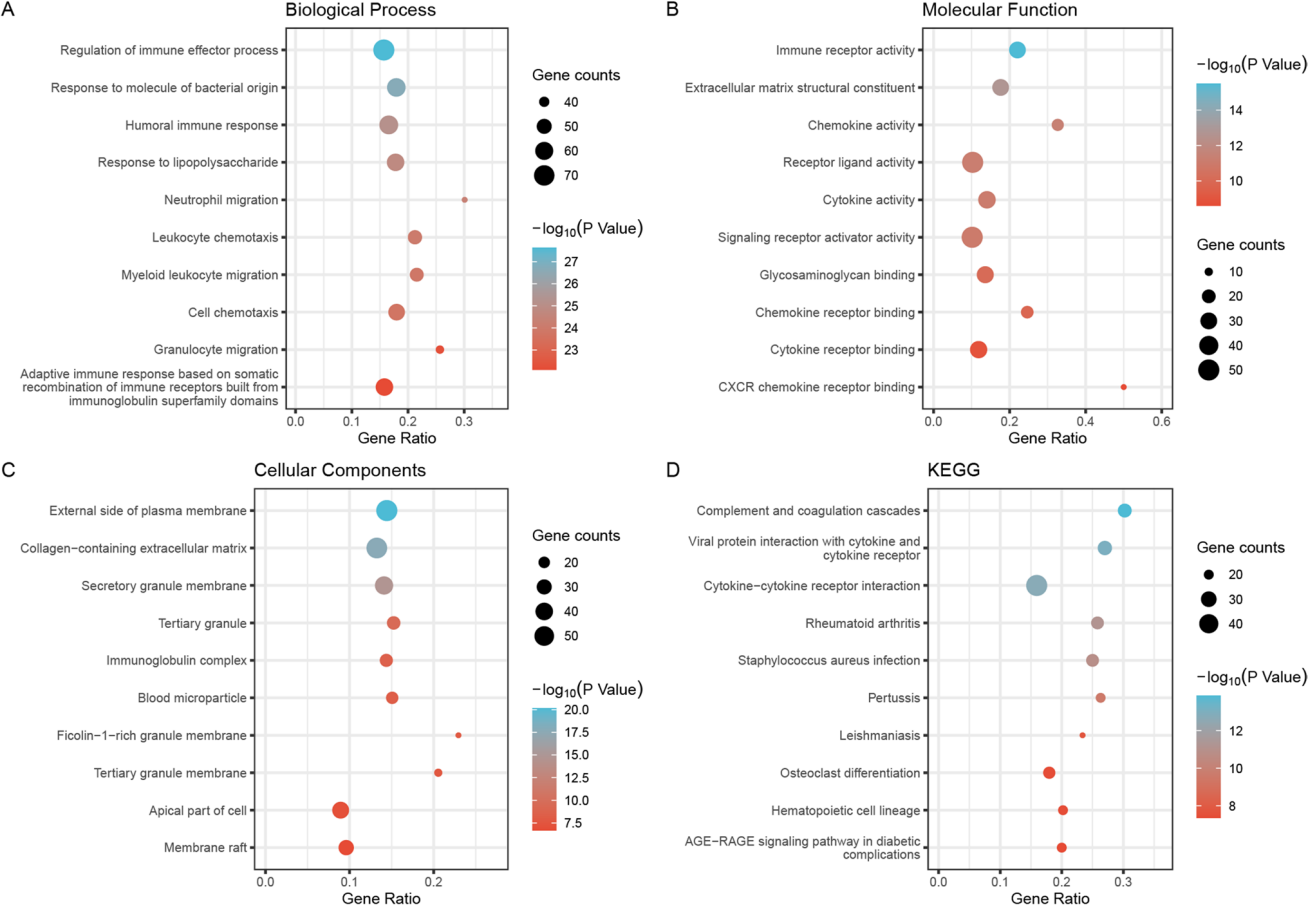
GO and KEGG enrichment analysis (top 10), figure (**A**), (**B**), (**C**), (**D**) show the enrichment analysis results of differentially expressed genes between active and inactive groups in biological processes, molecular functions, cellular components, and KEGG pathway respectively, the vertical coordinates exhibit the 10 enriched functional regions, the horizontal coordinates exhibit the proportion of genes, and the bubble size indicate the number of genes enriched in GO function and KEGG pathway

GO and KEGG pathway analyses were carried out to gain further insight into the function of DEGs in active and inactive phases of UC. Results of GO analysis revealed that DEGs were enriched in biological processes related to “regulation of immune effector process”, “response to molecule of bacterial origin”, “humoral immune response”, “response to lipopolysaccharide”, as presented in Fig. 3-A. Furthermore, the analysis of molecular function demonstrated that DEGs were chiefly involved in “immune receptor activity”, “extracellular matrix structural constituent”, “chemokine activity”, “receptor ligand activity”, as depicted in Fig. 3-B. Similarly, cellular components analysis showed that DEGs were enriched in multiple processes, including “external side of plasma membrane”, “collagen-containing extracellular matrix”, “secretory granule membrane”, “tertiary granule”, as shown in Fig. 3-C. Additionally, GO analysis identified DEGs related to regulating immune, inflammatory and metabolic processes in UC between the active and inactive phases. KEGG results revealed that DEGs were primarily enriched in immune and inflammatory processes, such as “Complement and coagulation cascades”, “Viral protein interaction with cytokine and cytokine receptor”, “Cytokine-cytokine receptor interaction”, “Rheumatoid arthritis”, as presented in Fig. 3-D.

### GSEA analysis of immunological signature gene set

**Fig. 4:**
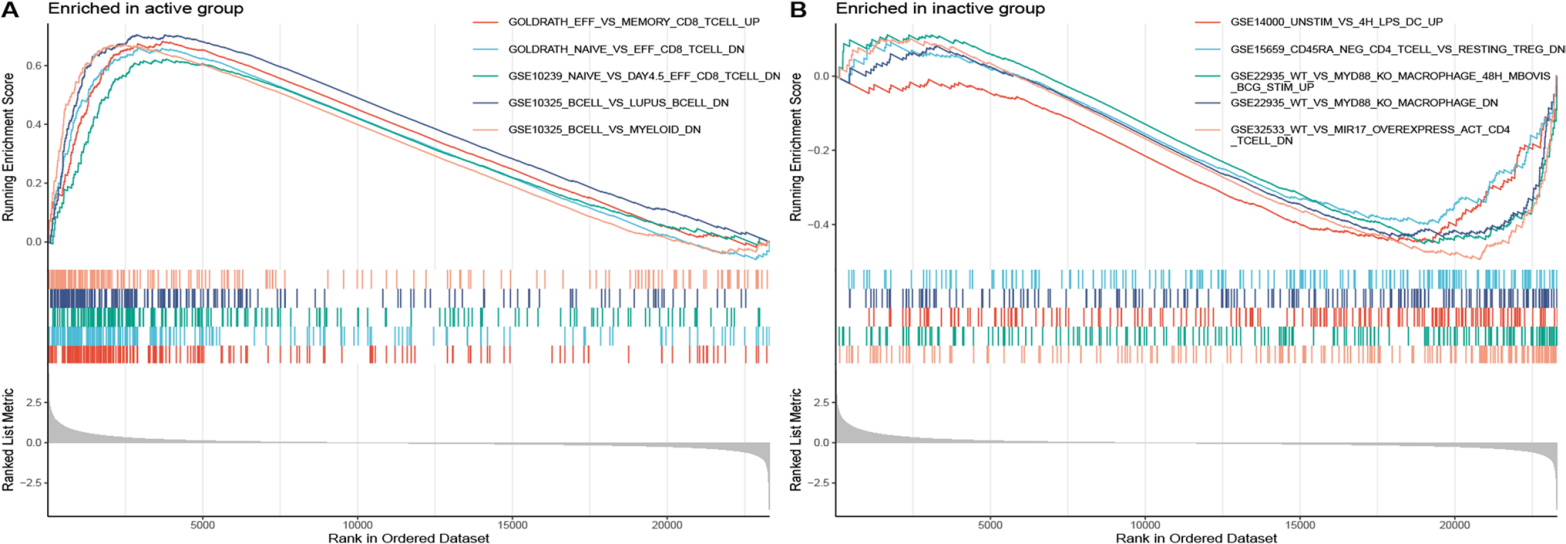
GSEA of immunological signature gene set

To explore the potential mechanism of immunological regulation during the progression from inactive to active condition, the immunological signature gene set from MsigDB database served as the reference for GSEA of DEGs. 2388 significantly enriched gene sets were figured out with the screening criteria of “|normalised enriched score (NES)|> 1 and q-value < 0.05”. The active group showed gene sets enriched mainly in CD8 T cells, B cells, and CD4 T cells. In contrast, gene sets significantly enriched in CD4 T cells, Macrophage and dendritic cells (DC) were observed in the inactive group. Table 1 shows the top 10 gene sets with the highest NES scores. The results suggest that immunological signature gene sets are implicated in UC under active and inactive conditions respectively, as illustrated in Fig. 4.

**Table 1:** Top 10 significant immunological signature enriched by DEGs in GSEA (NES > 0 group and NES < 0 group)

Gene set name	NES	p-value	p.adjust	q-values
GOLDRATH_EFF_VS_MEMORY_CD8_TCELL_UP	2.315	1.00E-10	1.40E-09	6.25E-10
GOLDRATH_NAIVE_VS_EFF_CD8_TCELL_DN	2.234	1.00E-10	1.40E-09	6.25E-10
GSE10239_NAIVE_VS_DAY4.5_EFF_CD8_TCELL_DN	2.110	1.00E-10	1.40E-09	6.25E-10
GSE10325_BCELL_VS_LUPUS_BCELL_DN	2.391	1.00E-10	1.40E-09	6.25E-10
GSE10325_BCELL_VS_MYELOID_DN	2.285	1.00E-10	1.40E-09	6.25E-10
GSE10325_CD4_TCELL_VS_BCELL_DN	2.444	1.00E-10	1.40E-09	6.25E-10
GSE10325_CD4_TCELL_VS_LUPUS_CD4_TCELL_DN	2.118	1.00E-10	1.40E-09	6.25E-10
GSE10325_CD4_TCELL_VS_MYELOID_DN	2.379	1.00E-10	1.40E-09	6.25E-10
GSE10325_LUPUS_BCELL_VS_LUPUS_MYELOID_DN	2.145	1.00E-10	1.40E-09	6.25E-10
GSE10325_LUPUS_CD4_TCELL_VS_LUPUS_BCELL_DN	2.467	1.00E-10	1.40E-09	6.25E-10
GSE32533_WT_VS_MIR17_OVEREXPRESS_ACT_CD4_TCELL_DN	-1.908	2.97E-07	2.29E-06	1.02E-06
GSE22935_WT_VS_MYD88_KO_MACROPHAGE_48H_MBOVIS_BCG_STIM_UP	-1.743	1.63E-05	8.53E-05	3.80E-05
GSE14000_UNSTIM_VS_4H_LPS_DC_UP	-1.725	1.85E-05	9.54E-05	4.25E-05
GSE22935_WT_VS_MYD88_KO_MACROPHAGE_DN	-1.677	< 0.001	< 0.001	< 0.001
GSE15659_CD45RA_NEG_CD4_TCELL_VS_RESTING_TREG_DN	-1.539	0.001	0.002	0.001
GSE41867_DAY6_VS_DAY15_LCMV_CLONE13_EFFECTOR_CD8_TCELL_UP	-1.600	0.001	0.003	0.001
GSE6259_DEC205_POS_DC_VS_BCELL_UP	-1.588	0.001	0.003	0.001
GSE19888_ADENOSINE_A3R_ACT_VS_TCELL_MEMBRANES_ACT_AND_A3R_INH_PRETREAT_IN_MAST_CELL_DN	-1.552	0.001	0.003	0.001
GSE37301_HEMATOPOIETIC_STEM_CELL_VS_PRO_BCELL_DN	-1.560	0.001	0.004	0.002
GSE9960_GRAM_NEG_VS_GRAM_NEG_AND_POS_SEPSIS_PBMC_DN	-1.520	0.001	0.004	0.002

*DEG* differentially expressed genes, *GSEA* gene set enrichment analysis, *NES* normalized enriched score

### Analysis of immune cell infiltration

**Fig. 5:**
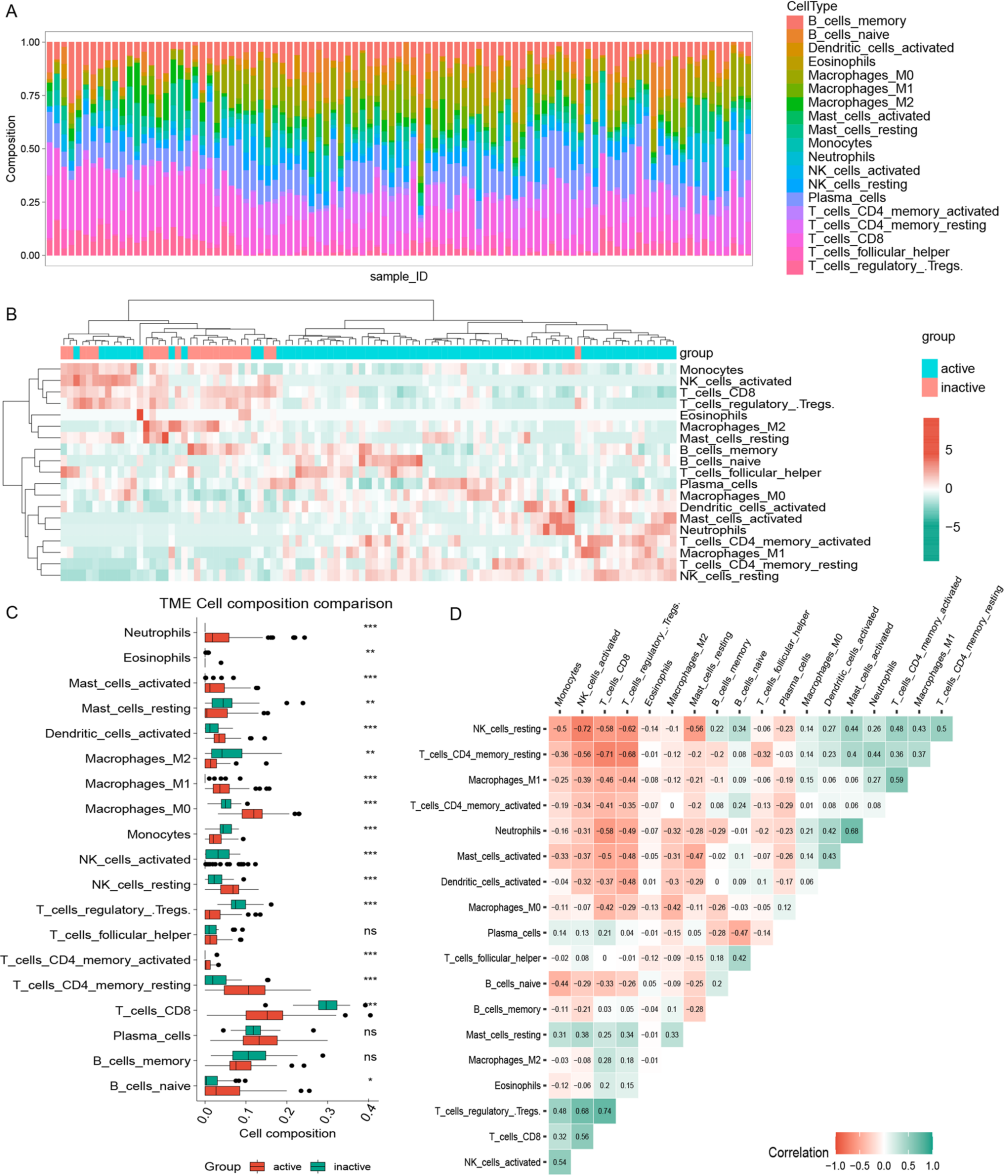
**A** The immune infiltration scores of the samples, the horizontal coordinates are the sample numbers, the different colors of the bars represent different immune cell types, and the size of the area of the various colors represents the proportion of different immune cell infiltration to the total immune cell infiltration; **B** The heat map of each sample with immune cell infiltration scores between the active and inactive groups; **C** The comparison of various immune infiltration scores between the active and inactive samples, in which red and green bars represent active and inactive patients, respectively; **D** The correlation analysis of various immune cells in the active phase, in which the red area represents negative correlation, green area represents positive correlation

Analysis of immune cell infiltration revealed differences between patients with active and inactive UC (Fig. 5-A, B). The Wilcoxon test demonstrated a significant increase in the infiltration of neutrophils, M0 Macrophages, M1 Macrophages, resting CD4 T memory cells, and activated memory CD4 T cells in the active group. In contrast, memory B cells, CD8 T cells, and regulatory T cells (Tregs) infiltration was significantly higher in inactive group, as shown in Fig. 5-C. Figure 5-D displays the correlation analysis among different immune cells in the active group, which shows that CD8 T cells was positively correlated with regulatory T cells (cor = 0.739; P < 0.001), neutrophils was positively correlated with activated mast cells (cor = 0. 0.683; P < 0.001), and CD8 T cells was negatively correlated with Macrophages_M0 (cor = -0.423; P = 0.003).

### Identification of key modules via WGCNA

**Fig. 6:**
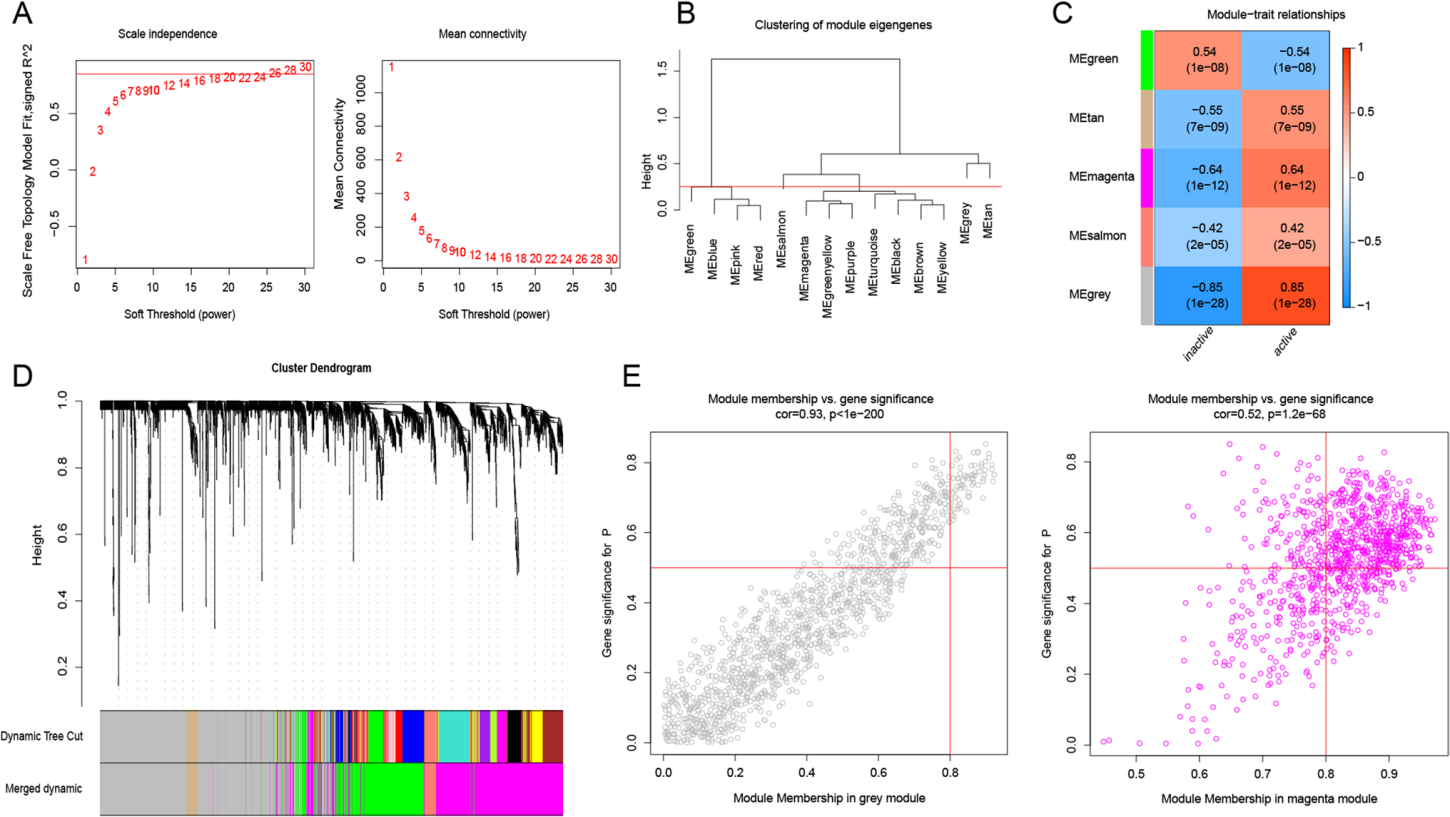
Weighted gene co-expression network analysis. **A** Relationship between soft thresholds and scale-free fit indices (left), and mean connectivity (right); **B** Dendrogram of the modules (merged with height = 0.25); **C** Correlation of module specified genes with active and inactive phases. **D** Gene dendrograms based on variance measurement (1-TOM) and merged modules figure; **E** Scatter plots of MM and GS of significant module genes of the key module

Module identification was conducted using the WGCNA algorithm to identify the key genes involved in the transition process between active-inactive conditions. Among the top 3000 genes with high standard deviations in the GSE75214 dataset, 14 gene co-expression modules were created using a power parameter of 26 (Fig. 6-A). The modules were subsequently merged with a height parameter set at 0.25, resulting in the identification of 5 modules (Fig. 6-B, C, D). Based on the *p*-value, the gray module (cor = -0.85; P = 1e-28) and magenta module (cor = -0.64; P = 1e-12) were found to be closely related to the active and inactive phases of UC, respectively. As a result, the gray and magenta modules, which comprised a total of 2208 genes, were selected for subsequent analysis.

### Acquisition of herb-compound-target network and identification of candidate genes for enrichment analysis

**Fig. 7:**
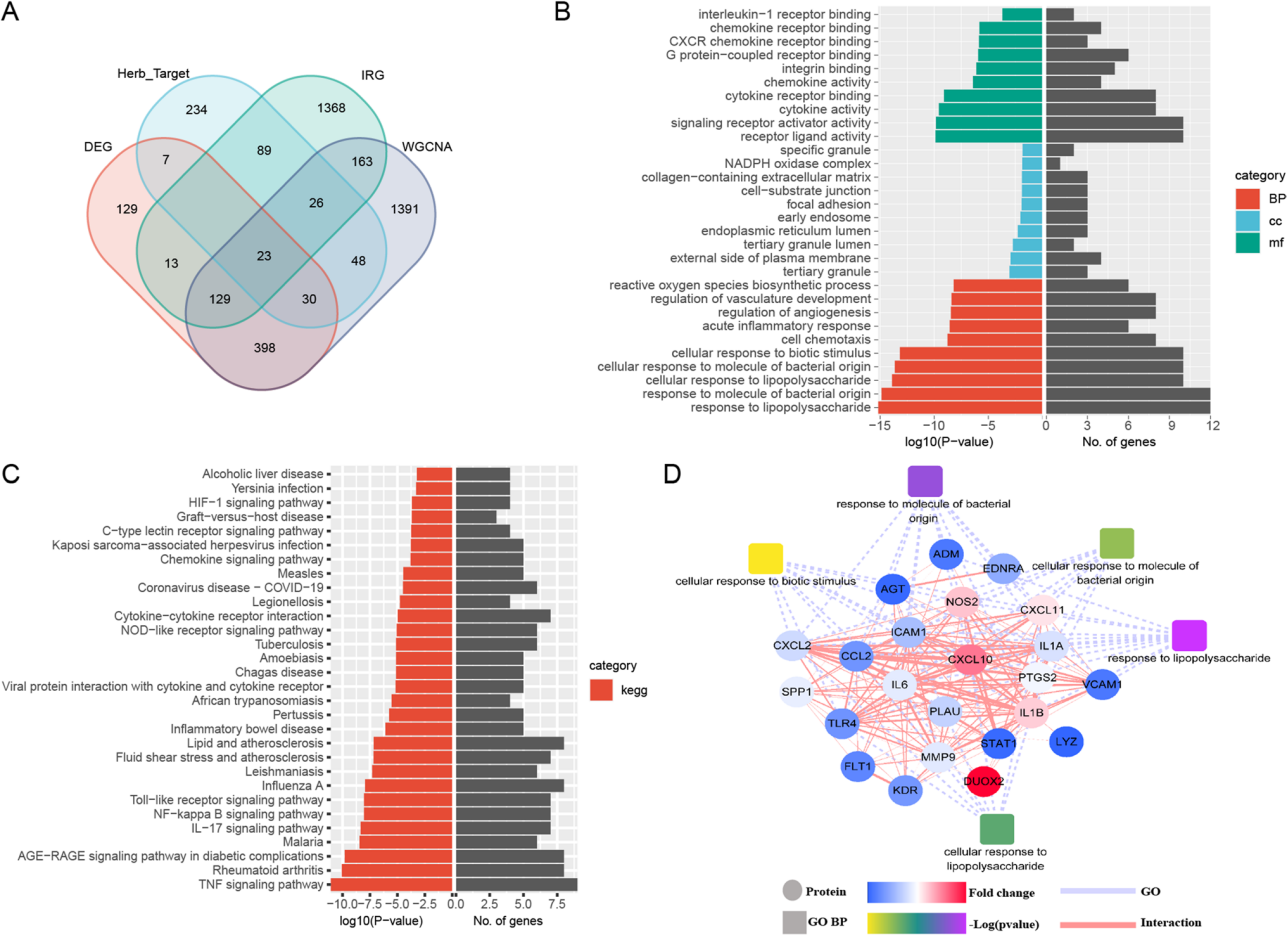
Functional analysis of key candidate genes. **A** candidate genes obtained by Venn diagram; **B** GO analysis of candidate genes, different colors represent different types of BP, CC, MF item; **C** KEGG enrichment of the candidate genes; **D** The PPI network of the candidate genes, the round nodes represent target proteins, quadrate nodes represent GO_BP items, the colors on the target nodes represent the fold-chang (FC) values, blue indicates lower FC, white is moderate, and red indicates higher FC, different colors of GO_BP nodes represent different -log (p-value)

The herb-compound-target network was obtained from the TCMSP database. To ensure the reliability of the network, compounds with oral bioavailability (OB) ≥ 30% and drug likeness (DL) ≥ 0.18 were selected, resulting in a total of 464 herbal targets after matching in the STRING database. To identify candidate targets for treating active UC by the means of herbal medicine via regulating immune function, DEG, IMM, WGCNA and herbal medicine targets were intersected and 23 intersecting genes were identified, as displayed in Fig. 7-A. The candidate genes were subjected to gene ontology (GO) and KEGG pathway enrichment analysis. GO analysis (Fig. 7-B) revealed that the candidate genes are mainly involved in biological processes related to immunity and inflammation, including “response to lipopolysaccharide”, “response to molecule of bacterial origin”, “cellular response to lipopolysaccharide”, “cellular response to molecule of bacterial origin”, “cellular response to biotic stimulus”. KEGG enrichment analysis (Fig. 7-C) revealed that the candidate genes were primarily enriched in the pathways of “TNF signaling pathway”, “Rheumatoid arthritis” and “IL-17 signaling pathway”. Finally, the key genes of the candidate targets were analyzed using the protein–protein interaction (PPI) network (Fig. 7-D).

### Screening of hub genes by machine learning

In order to obtain the core targets with impact significance on active and inactive phases, three different algorithms (LASSO, SVM-RFE and Random Forest) were applied to further screen the 23 candidate genes.

**Fig. 8:**
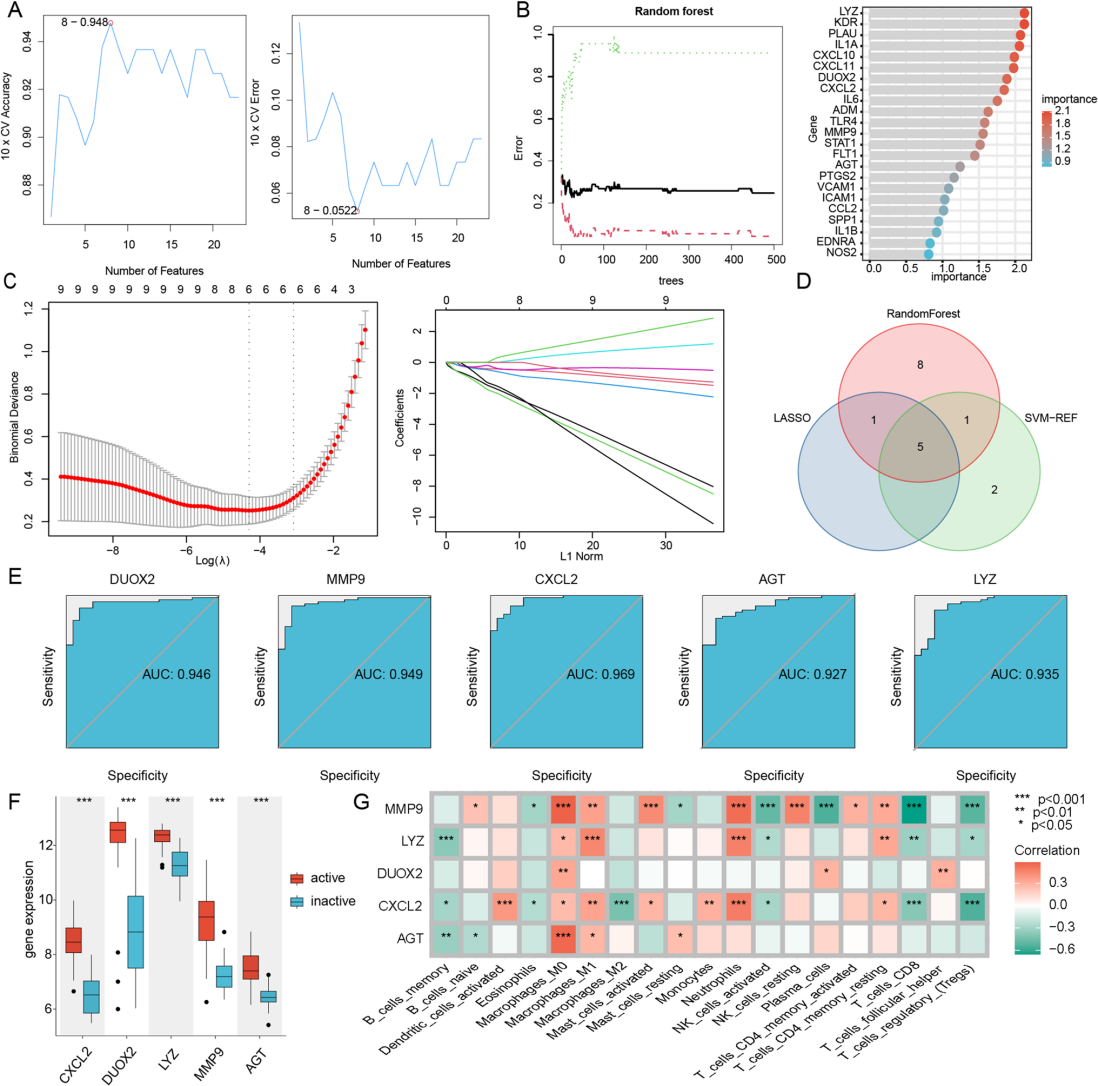
Screening of hub genes by machine learning. **A** The model was found to have the best accuracy and the lowest error rate when the number of genes was 8 by the SVM-RFE algorithm; **B** Important genes were obtained by the Random Forest (RF) algorithm, and the error rate leveled off with the increase in the number of decision trees, while the importance of the 23 key genes was ranked; **C** Cross-validation error path diagram (tenfold cross validation) and Coefficient Path diagram of LASSO algorithm, the selection result of the optimal parameter λ and the acquisition of 5 genes; **D** Combination of three machine learning algorithms to obtain hub targets; **E** The roc curves of the 5 hub genes; **F** Expression of the 5 hub genes between active and inactive groups, where *** represents p-value < 0.001; **G** Heatmap of the correlation between the 5 hub genes and immune infiltrating cells. SVM-RFE: support vector machine—recursive feature elimination; LASSO: Least absolute shrinkage and selection operator; roc: receiver operating characteristic curve

Based on SVM-RFE algorithm, the optimal hub genes were identified through tenfold cross-validation, and the results are displayed in Fig. 8-A, which shows the error rate plot and the correct rate plot for tenfold cross-validation (10 × CV) of the model. By the points marked as red circles, we determined that the algorithm has the smallest 10 × CV error and the best 10 × CV accuracy when 8 genes are selected.

The 23 key genes described above were entered into a random forest classifier for analysis. Figure 8-B shows that as the number of decision trees in the forest increases, the misclassification rate decreases rapidly and stabilizes after a period of fluctuation. The black, red and blue lines in the figure represent the variation in the error rate with the number of decision trees for all individuals, the active period sample, and the inactive sample, respectively. The error rate rapidly decreases and stabilizes after a period of fluctuation as the number of decision trees in the forest increases. The figure show that the overall error rate reaches its lowest around 100 decision trees and remains stable after after more than 120 trees. The right part of Fig. 8-B also demonstrates the importance of each gene in influencing the results. We chose 1.2 as the significance screening threshold from which we identified 15 genes.

subsequently, we also characterized these 23 genes with LASSO regression analysis and tenfold cross validation. Figure 8-C shows the coefficient curves and the optimal selection of parameters, and the optimal lambda value (0.01637392), was selected by "lambda.min". Finally, we identified 6 genes with non-zero coefficients, which were closely related to the active and inactive phases of UC, as shown in Fig. 8-C.

Five hub genes including CXCL2, DUOX2, LYZ, MMP9, AGT were obtained based on the common hub genes identified via the 3 machine learning algorithms, which may become the key gene targets for TCM to promote the UC from active to inactive phase based on the regulation of immune mechanisms (Fig. 8-D).

To further validate the diagnostic value of CXCL2, DUOX2, LYZ, MMP9, and AGT on whether the UC is in the active or inactive phase, receiver operating characteristic (ROC) analysis was performed and the area under the curve (AUC) of ROC was assessed: DUOX2 (AUC:0.946), MMP9 (AUC:0.949), CXCL2(AUC:0.969), AGT(AUC:0.927), and LYZ(AUC:0.935), as shown in Fig. 8E. The results suggested a high rate of correctness of all the 5 hub genes, which also imply that these 5 hub genes are key molecules for promoting the transition of active UC to the inactive states. Besides, according to the data in the GSE75214 dataset, it is found that these 5 hub genes are highly expressed in active phase of UC, as shown in Fig. 8F. Correlation analysis of the five hub genes with the immune infiltration scores revealed that MMP9 was positively correlated with Macrophages_M0 (cor = 0.585, P = 8.34E-08), AGT was positively correlated with Macrophages_M0 (cor = 0.564, P = 2.78E-07), and MMP9 was positively correlated with Neutrophils (cor = 0.544, P = 5.31E-07). Meanwhile, MMP9 was negatively correlated with T_cells_CD8 (cor = -0.657; P < 0.001), MMP9 was negatively correlated with Plasma_cells (cor = -0.547, P = 7.47E-07), and CXCL2 was negatively correlated with T_cells_regulatory_(Tregs) (cor =—0.542, P = 5.98E-07). These results further demonstrate the critical role played by these immune cells in the progression of UC between active and inactive phases.

### Obtaining of the functional herbs

**Fig. 9:**
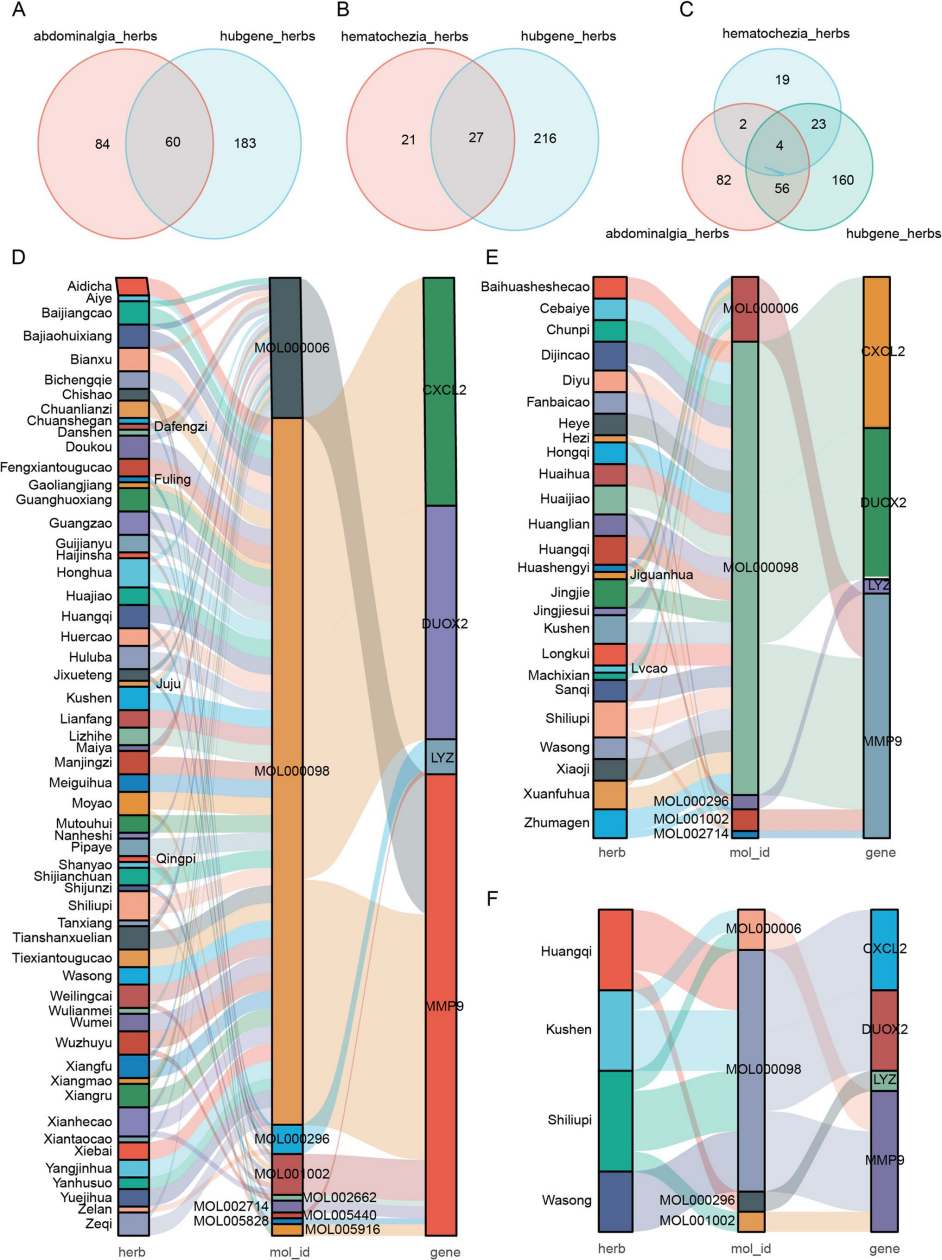
**A** The intersection of hub gene herb and abdominal pain herbal medicine; **B**: the Venn diagram of the intersection of hub gene-associated herb and hematochezia-associated herbal medicine; **C**: Venn diagram of the intersection of hub gene-associated herb, abdominal pain related herbal medicine and hematochezia-related herbal medicine; **D**, **E**, and **F** are Network relationship between compounds, targets and intersection herbs (obtained from **(A)**, **(B)**, and **(C)** respectively

Five hub targets were retrieved from the TCMSP database, and small molecules meeting OB ≥ 30 and DL ≥ 0.18 criteria, correlating to 243 herbs with potential immune-modulating function, were identified. These herbs were thought to have the ability to transform UC from an active to an inactive phase. To figure out herbs capable of treating active ulcers in the active phase as well as alleviating the symptoms of UC, a total of 144 herbs associated with abdominal pain and 48 herbs associated with hematochezia were identified from the Symmaps database. The overlap between the 243 hub genes related herbs and the two aforementioned symptoms associated herbs was shown in Fig. 9 A-C; the network between herb, molecule, hub target was constructed as shown in Fig. 9 D-F. Nevertheless, the hub gene- AGT identified in this part failed to correlate with abdominal pain and hematochezia. A total of nine molecules were involved in the 3 herb-molecule-target networks, which is shown in Table 2.

**Table 2:** The information of 9 molecules involved in the 3 herb-molecule-target networks

mol_id	molecule_name	OB	DL	SMILES
MOL000098	quercetin	46.43	0.28	C1(= CC2 = C(C(= C1)O)C(= O)C(= C(O2)C1 = CC(= C(C = C1)O)O)O)O
MOL001002	ellagic acid	43.06	0.43	C1(= CC2 = C3C(= C1O)OC(= O)C1 = C3C(= C(C(= C1)O)O)OC2 = O)O
MOL000006	luteolin	36.16	0.25	C12 = CC(= CC(= C1C(= O)C = C(O2)C1 = CC = C(C(= C1)O)O)O)O
MOL002714	baicalein	33.52	0.21	C12 = CC(= C(C(= C1C(= O)C = C(O2)C1 = CC = CC = C1)O)O)O
MOL000296	hederagenin	36.91	0.75	O[C@@H]1CC[C@]2(C(= CC[C@@H]3[C@H]4CC[C@H]([C@@H](CC[C@@H](CC)C(C)C)C)[C@@]4(C)CC[C@@H]23)C1)C
MOL002662	rutaecarpine	40.30	0.60	O = C1N2CCC3 = C(NC4 = CC = CC = C34)C2 = NC2 = CC = CC = C12
MOL005440	Isofucosterol	43.78	0.76	O[C@@H]1CC2 = CC[C@H]3[C@H]4[C@@]([C@H](CC4)[C@@H](CC/C(= C/C)/C(C)C)C)(CC[C@@H]3[C@]2(CC1)C)C
MOL005916	irisolidone	37.78	0.30	C1(= CC2 = C(C(= C1OC)O)C(= O)C(= CO2)C1 = CC = C(C = C1)OC)O
MOL005828	nobiletin	61.67	0.52	C12 = C(C(= C(C(= C1OC(= CC2 = O)C1 = CC = C(C(= C1)OC)OC)OC)OC)OC)OC

### Molecular docking results

**Fig. 10:**
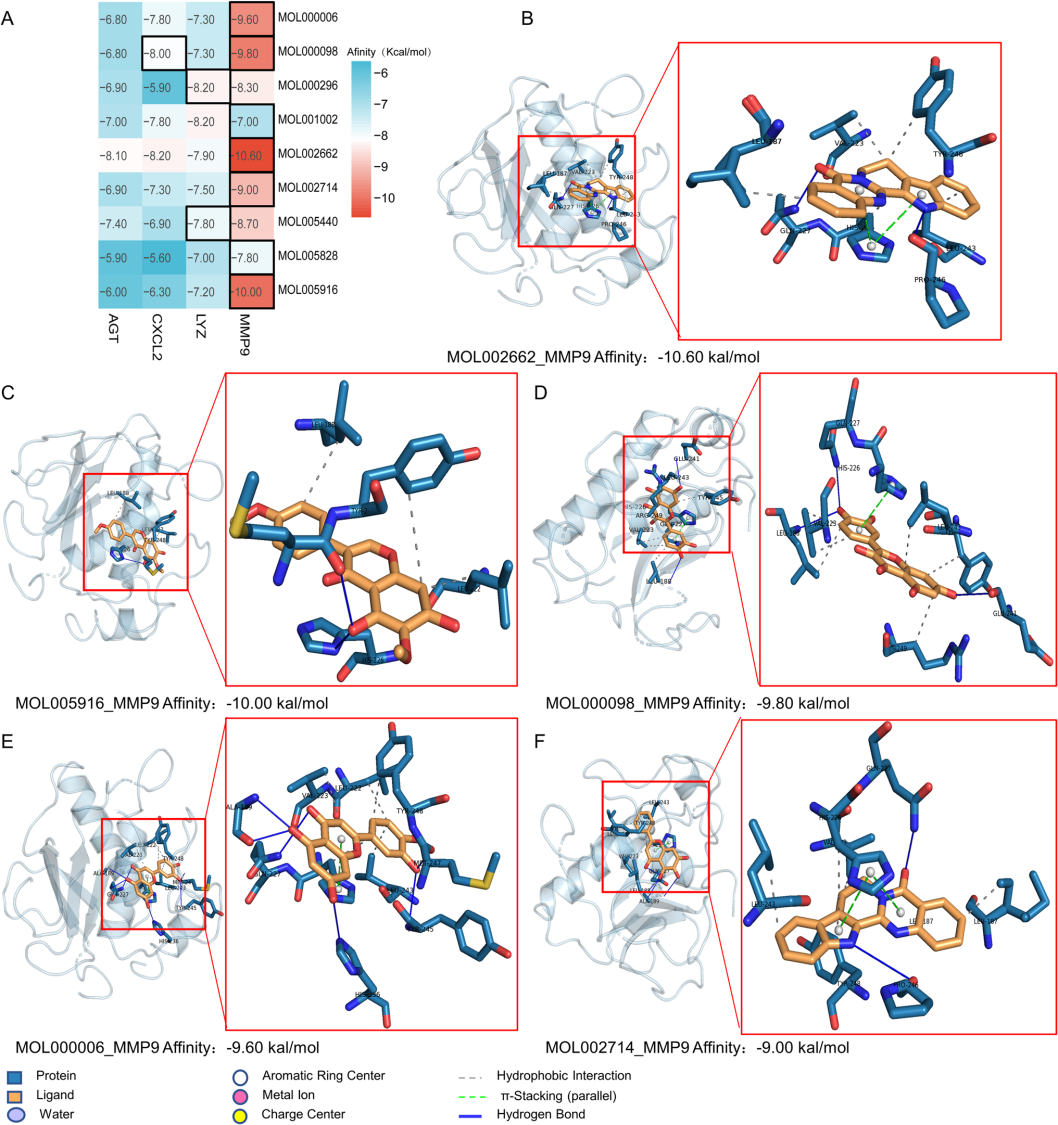
Molecular docking model. **A** The heat map of the molecular docking results of the core compound with the target, black squares indicate the exist of herb-compound-target network. Figure **(B-F)** The docking model of small molecule ligand and protein: **B** MOL002662-MMP9; **C** MOL005916-MMP9; **D** MOL000098-MMP9; **E** MOL000006-MMP9; **F** MOL002714-MMP9. The red boxes show the enlarged pictures of the compound-target complexes

Four kinds of proteins were identified by inputting the 5 hub genes into the PDB database. Molecular docking of 9 potential TCM small molecules with the proteins obtained above revealed that the docking scores of small molecule-target combinations present in the three networks in step 8 (TCM prediction) ranged from -7.0 to -10.6 kcal/mol. The heat map of docking scores of the 9 core compound with hub target is shown in Fig. 10A. The results indicate that the MMP9 had better docking scores, and the molecule could embed in the binding pocket of the target and build π-stacking and hydrogen bond with a number of residues around the target, as shown in Fig. 10 B-F, all of these findings suggest that the TCM small molecule compounds are capable of matching with the hub targets preferably and exhibit excellent binding force, indicating active binding between the complexes. Furthermore, the molecule-target combinations weren’t included in the herb-molecule-target network also showed remarkable binding potential, such as LYZ-MOL001002 (affinity: -8.2 kcal/mol) and CXCL2-ML002662 (affinity: -8.2 kcal/mol). In addition, AGT, which was not associated with the symptomatic herb, showed advantageous binding potential with MOL002662 (affinity: -8.1 kcal/mol), suggesting that there may be other herbs and small molecules associated with the treatment of UC which are worth to explore.

### Molecular dynamic simulation

**Fig. 11:**
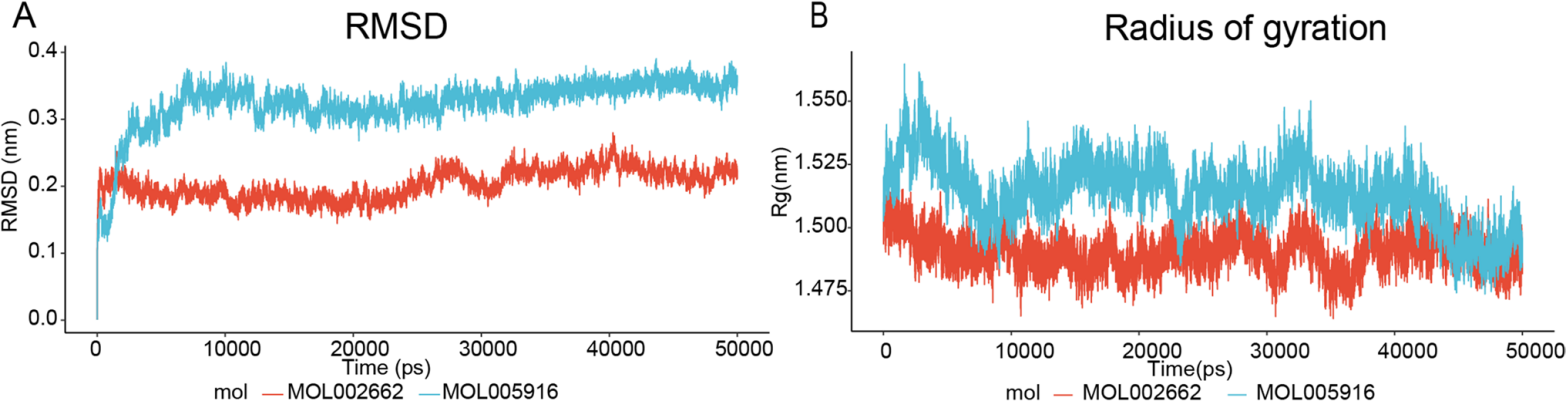
Results of molecular dynamic simulations. **A** RMSD of MOL002662-MMP9, MOL005916- MMP9; **B** The radius of gyration of two complexes

The top two complexes with the lowest affinity scores (MOL002662-MMP9 and MOL005916-MMP9) were subjected to molecular dynamic simulations. During the 50 ns of molecular dynamic simulation, the C-α backbone of the complexes rapidly stabilized. The RMSD values for MOL002662 and MOL005916 showed fluctuations with small differences, with ranges of 0.3–0.4 nm and 0.15–0.25 nm, respectively (Fig. 11-A). The radius of gyration reflects the density of the protein structure, with a smaller variation in denser structures. Both simulations demonstrated stable radius of gyration with values ranging from 1.464 to 1.565 nm (Fig. 11-B). Consistent radius of gyration throughout the simulations indicated excellent stiffness of the system. Hence, the MOL002662-MMP9 and MOL005916-MMP9 systems demonstrated stable structures in a balanced state.

## Discussion

In the pathogenesis of UC, a variety of immune cells, cytokines and chemokines are involved. This study identified several up-regulated genes that are significantly enriched in immune regulation, both in the active and inactive phases of UC. Furthermore, this study confirmed significant differences in infiltration of different types of immune cells such as M0 macrophage, M1 macrophage, neutrophils, activated memory CD4 T cells and B cells between the two groups, suggesting that both innate and adaptive immune responses are involved in the remission mechanism of UC. Evidence suggests that that some vital ingredients in TCMs are capable of regulating immune dysregulation, such as Treg/Th17 cells and M1/M2 polarization in the colonic mucosa of UC, which can help improve intestinal inflammation. In clinic practice, TCMs are effective in treating UC, with negligible toxicity and excellent tolerability. Nevertheless, the mechanism of action for most TCMs in regulating immune dysregulation between active and inactive UC remains uncertain. The findings of this study facilitate the prediction of core targets of TCMs that have the potential to coordinate immune dysregulation of UC through immunomodulatory pathways via machine learning. This can lead to identification of immunomodulatory TCMs that can be helpful for treating UC.

The present study identified 5 key targets, namely MMP9, LYZ, CXCL2, AGT, DUOX2, that TCM targets to promote active UC transit into inactive condition through immune reaction modulation. MMP9, a protease that is capable of degrading various proteins in the extracellular matrix, is upregulated in UC, inducing pro-inflammatory cytokines, which is considered a crucial target for the treatment of UC (O'Sullivan et al. 2017). Currently, a variety of herbal formulas have been shown to be able to suppress the activity of MMP9, which can help to alleviate intestinal inflammation and restore the integrity of the intestinal mucosal (Wen et al. 2021). DUOX2 is capable of promoting the production of H_2_O_2_ from NADPH, which enters the intestinal epithelial mucus layer and plays a critical role in the innate immune defense of the intestinal epithelial barrier (Taylor and Tse 2021). In addition, DUOX2 has been identified both as a risk gene for inflammatory bowel disease (IBD) (MacFie et al. 2014) and an essential host factor for maintaining gut microbiota homeostasis (Grasberger et al. 2021). CXCL2 is an inflammatory chemokine that recruit leukocytes and other immune cells to the site of infection during the immune response, producing anti-inflammatory effects in case of UC (Wen et al. 2021). Angiotensinogen is involved in the production of angiotensin II, which is a major target of the renin-angiotensin system (RAS). Although there is no evidence showing angiotensin is associated with UC, studies have reported that it is associated with several immune cells, including B cells, CD4 + T cells, and macrophages, and its expression values is associated with markers such as NK, TAM, and Treg cells (Wu et al. 2022). This is consistent with the results of the present study, which identified angiotensin involved in the regulation of immune reactions. Furthermore, AGT is a critical regulator of apoptosis in intestinal epithelial cells (Wang et al. 2013). In summary, these findings suggest that angiotensinogen may play a role in the pathogenesis of IBD through the regulation of immune responses. Lysozyme (LYZ) is a major innate immune protein in the human body. Targeted destruction of panniculocyte lysozyme (Lyz1) has been shown to alleviate the damage caused by colitis in mice, making it a potentially significant regulator of intestinal anti-inflammatory and pro-inflammatory reactions and therefore a crucial target for the treatment of UC (Yu et al. 2020). Overall, the 5 core targets identified in the present study are believed to be crucial for the treatment of UC.

In damaged tissues, monocytes differentiate into M1 or M2 macrophages. M1 macrophages are induced primarily by bacterial pathogens and are responsible for phagocytosis, bacterial killing, and release of TNF-α, IL-1β, and IL-6, which promotes inflammatory reactions. M2 macrophages, on the other hand, mainly release anti-inflammatory factors, which weaken the immune response, inhibit inflammation, and promote tissue repair (Locati et al. 2020). There is evidence of dysregulation of M1/M2 macrophage polarization in colonic lesions of ulcerative colitis patients (Dharmasiri et al. 2021). Moreover, it has been founded that macrophage M1 are significantly enriched in the inflamed bowel while M2 macrophages are replete in the non-inflamed ileum. Results from this study revealed that M0 and M1 macrophage infiltration was more pronounced in the active UC intestinal mucosa compared to the inactive phase; on the other hand, M2 macrophage infiltration was more pronounced in the inactive phase, which is consistent with previous research (Dharmasiri et al. 2021). The difference in immune cell infiltration suggests that regulating M1/M2 macrophage polarization may help maintain UC remission (Dharmasiri et al. 2021), which is an effective treatment for UC (Lv et al. 2021). The therapeutic targets identified in this study, including MMP9, LYZ, CXCL2, and AGT, were closely associated with cell infiltration levels of M1 macrophages in active UC mucosa. Furthermore, CXCL2 was negatively correlated with cell infiltration levels of M2 macrophages, indicating that TCMs can improve levels of cellular infiltration for M1 and M2 macrophages by targeting MMP9, LYZ, CXCL2, and AGT.

Clinical research has demonstrated that individuals with ulcerative colitis (UC) tend to have a larger Th17 cell count and a smaller Treg cell count (Gong et al. 2016). Treg cells are known for their effectiveness in preventing inflammation in the colonic mucosa. However, interleukin-6 (IL-6) and/or interleukin-23 (IL-23) have the capability to cause the conversion of Treg cells into Th17 cells, which can then secrete pathogenic and pro-inflammatory cytokines such as IL-17 and TNF-α that play important roles in intestinal inflammation (Tsun et al. 2011). The currently available research indicates that the disparity between Th17 and Treg cells' quantities may play a significant role in the onset, progression, and intensity of UC (Ueno et al. 2018). The conclusion substantiated by the results of this study is that the expression levels of hub target genes MMP9 and CXCL2 were negatively correlated with the number of Treg cells during an active case of UC. This observation indicates that the TCMs identified in this study could restore the balance of Th17/Treg cells resulting in the remission of UC.

The model constructed in the present study predicted that TCMs such as Hedysarum Multijugum Maxim., can act on UC and aid in the transition from active to inactive UC. These TCMs can also alleviate clinical symptoms such as hematochezia and abdominal pain. Besides, Astragaloside IV, an extract of Astragalus, has been found to aid in macrophage re-polarization, reduce the production of pro-inflammatory cytokines, and increase the production of anti-inflammatory cytokines, thus alleviating experimental dextran sulphate sodium-induced UC (Tian et al. 2021). In addition, studies have indicated that Bitter Ginseng is safer for UC patients and has a higher clinical remission rate compared to 5-aminosalicylic acid (Chen et al. 2020). Furthermore, researches have indicated that Bitter Ginseng is safer for UC patients. The component oxymatrine, found in Bitter Ginseng, has been shown to improve the imbalance of Treg and Th17 cells, thereby achieving both immunosuppressive and anti-inflammatory effects (Ma et al. 2017). Meanwhile, Pomegranate Polyphenols extract has been proven to alleviate colitis (Kim et al. 2017), and further research could explore the use of Pomegranate Peel and Cotyledon Fimbriata Turcz. (Wasong) in treating active UC.

## Conclusion

The study indicates that there are significant differences in immune cell infiltration and immune-related genes between the active and inactive stages of UC. By utilizing machine learning and network pharmacology, this study has provided the first identification of 5 core therapeutic targets of TCMs that can facilitate the conversion of UC from the active to the inactive phase. This finding is based on the understanding that TCMs are effective in regulating the immune homeostasis of UC. The study also predicted that there are 243 herbs which can facilitate the active to inactive transfer, 60 herbs which can relieve abdominal pain in active UC, and 27 herbs which can relieve hematochezia in active ulcers. In addition, the study predicted that Hedysarum Multijugum Maxim. (Huangqi), Sophorae Flavescentis Radix (Kushen), Granati Pericarpium (Shiliupi), and Cotyledon Fimbriata Turcz. (Wasong) are four significant herbs that can relieve abdominal pain caused by active ulcers and hematochezia. Moreover, the study also identified novel candidate TCMs for relieving UC. Nonetheless, the study has some limitations because the compound-herb and compound-target network used in the study may not be comprehensive due to limitations in the information available in the database. Moreover, diverse clinical application methods may affect the effects of the herbs. Hence, the study's findings may be limited in their application field. To address this limitation, we recommend expanding the scope of the database screening to explore the role of the significant target components that alleviate active UC. Doing so will allow for the more comprehensive identification of candidate drugs and better exploration of the drugs' ability to alleviate UC and its interventional mechanism. Additionally, clinical trials are essential in elucidating the mechanism of action of drugs in alleviating UC. This knowledge is critical to develop effective treatments for UC.

### Supplementary Information

Below is the link to the electronic supplementary material.

Supplementary file1 (XLSX 6638 KB)

## Data Availability

We are grateful to the research team for providing us GSE75214 dataset. All data supporting the article is provided in this article.

## Abbreviations

TCM: Traditional Chinese Medicine
UC: Ulcerative colitis
IBD: Inflammatory bowel disease
DEGs: Differentially expressed genes
WGCNA: Weighted gene co-expression network analysis
IRG: Immune-related genes
HRTs: Herb related targets
PPI: Protein-protein interaction
GO: Gene ontology
KEGG: Kyoto Encyclopedia of Genomes
BP: Biological process
CC: Cellular component
MF: Molecular function
OB: Oral bioavailability
DL: Drug likeness
HIPL: Protein-Ligand Interaction Profiler
RMSD: Root mean square deviation
RMSF: Root mean square fluctuations
